# Constitutive STAT5 phosphorylation in CD34^+^ cells of patients with primary myelofibrosis: Correlation with driver mutation status and disease severity

**DOI:** 10.1371/journal.pone.0220189

**Published:** 2019-08-01

**Authors:** Carlotta Abbà, Rita Campanelli, Paolo Catarsi, Laura Villani, Vittorio Abbonante, Melania Antonietta Sesta, Giovanni Barosi, Vittorio Rosti, Margherita Massa

**Affiliations:** 1 Center for the Study of Myelofibrosis, Laboratory of Biochemistry, Biotechnology and Advanced Diagnosis, IRCCS Policlinico San Matteo Foundation, Pavia, Italy; 2 Laboratory of Biochemistry, Biotechnology and Advanced Diagnosis, IRCCS Policlinico San Matteo Foundation, Pavia, Italy and Department of Molecular Medicine, University of Pavia, Pavia, Italy; 3 Laboratory of Biochemistry, Biotechnology and Advanced Diagnosis, IRCCS Policlinico San Matteo Foundation, Pavia, Italy; Stanford University, UNITED STATES

## Abstract

Primary Myelofibrosis (PMF) is a myeloproliferative disorder associated with *JAK2*V617F, Calreticulin (*CALR*) indels, and *MPL*W515L/K mutations activating the tyrosine kinase JAK2 and its downstream signaling pathway. The nature of signaling abnormalities in primary cells from PMF patients is poorly understood, since most of the work has been performed in cell lines or animal models. By flow cytometry we measured constitutive and cytokine induced phosphorylation of STAT5, STAT3, and ERK1/2 in circulating CD34^+^ cells from 57 patients with PMF (20 with prefibrotic-PMF) and 13 healthy controls (CTRLs). Levels of constitutive and TPO induced p-STAT5, and IL6 induced p-STAT3 were higher in patients than in CTRLs. Constitutive p-STAT5 values were lower in *CALR* than homozygous *JAK2*V617F mutated CD34^+^ cells from PMF patients. Moreover, constitutive p-STAT5 and IL6 induced p-STAT3 values correlated directly with circulating CD34^+^ cell number/L, and inversely with the frequency of circulating CD34^+^ cells expressing CXCR4. Constitutive p-STAT5 values of CD34^+^ cells were also inversely correlated with hemoglobin levels. When the patients were divided according with presence/absence of *JAK2*V617F mutation, all the correlations described characterized the *JAK2*V617F+ patients with prefibrotic-PMF (P-PMF). In conclusion, increased constitutive p-STAT5 and IL6 induced p-STAT3 values in circulating CD34^+^ cells characterize patients with PMF. Constitutive p-STAT5 and IL6 induced p-STAT3 values correlate with circulating CD34^+^ cell number/L, the frequency of circulating CD34^+^ cells expressing CXCR4 and hemoglobin levels within the prefibrotic *JAK2*V617F^+^ patient population. Our data point toward a complex activation of STAT5-dependent pathways in the stem/progenitor cell compartment, that characterize the phenotypic diversity of PMF.

## Introduction

Primary Myelofibrosis (PMF) is a clonal disorder of the hematopoietic progenitor cells (HPCs) characterized by bone marrow (BM) fibrosis, increased number of peripheral blood (PB) CD34^+^ cells, splenomegaly, and increased risk of leukemic transformation [1].

PMF has been associated with driver mutations such as *JAK2*V617F, Calreticulin (*CALR*) indels, and *MPL*W515L/K [2]. JAK2 is a non-receptor tyrosine kinase that acts as an important signal transducer in cytokine signaling and promoting growth, survival, and differentiation of various cell types [3]. All the driver mutations activate the cytokine/receptor JAK2 pathway and its downstream signaling such as the signaling transducer and activator of transcription (STAT1, 3, and 5), phosphatidylinositol 3-kinase (PI3K)/ AKT/mTOR, and the MAPK/ extracellular signal-regulated kinase (ERK) pathways.

Despite their importance, the nature of signaling abnormalities in primary cells from myeloproliferative neoplasm (MPN) patients is poorly understood. A number of studies, performed mainly in animal models or using cell lines have reported the role of *JAK2* and *MPL* mutations in activating the STAT5, STAT3, PI3K/AKT, and RAS-MAPK-ERK pathways [4–6]; although relevant, these data may not be representative of the physiologic/pathologic environment in humans. On the other hand, the results of studies performed with patients are not conclusive, possibly due to both non homogeneous methods of analysis and patient populations [7, 8]. The evaluation of the signaling pathways that may be altered by the driver mutations characterizing PMF may help in understanding both the disease phenotype and the cell subset where possible abnormalities take place.

It has been reported an increased constitutive signaling of p-ERK in BM CD34^+^ HPCs and of p-STAT5 and p-STAT3 in BM CD34^-^ cells from patients with PMF; no significant correlation was described between these signaling pathways and mutant *JAK2* allele burden [9]. In addition, gene expression profile in granulocytes from a limited number of patients with PMF showed an increased expression of key signaling intermediates downstream of JAK2 (i.e. STAT5B) in homozygous *JAK2*V617F mutant patients [10]. *JAK2*V617F mutation was associated with significantly increased levels of phosphorylated STAT5 in hemopoietic cells, most marked in megakaryocytes, of patients with MPNs [11]. Abnormal nuclear megakaryocytic staining for p-STAT5 expression, previously associated with the *JAK2*V617F mutation, was also associated with *MPL*W515L/K mutation [12].

In this study we perform a flow cytometry assay to measure constitutive and cytokine induced phosphorylation of STAT5, STAT3, and ERK1/2 in circulating CD34^+^ cells from patients with PMF and healthy controls (CTRLs) comparable for age. We investigate the correlations of the phosphorylation patterns with mutated *JAK2*, *CALR*, and *MPL* genotypes and clinical disease parameters.

## Methods

### Study population

PB of 57 consecutive subjects with PMF was collected from the Center for the Study of Myelofibrosis, IRCCS Policlinico San Matteo Foundation, Pavia (Italy). All the patients included in the study gave a written informed consent for PB collection and participation in research studies related to their disease. We excluded subjects receiving disease-modifying drugs before or on the date of base-cohort entry (hydroxyurea, interferon, ruxolitinib, corticosteroids, immunosuppressive agents, and danazol). Diagnosis of overt PMF or prefibrotic-PMF (P-PMF) was based on review of BM biopsy samples by an expert pathologist according with the 2016 WHO criteria [13]. Data on medical history, concomitant diseases and drugs were obtained by interview and recorded in the data-base. Patients were assigned a prognostic score based on the International Prognostic Scoring System (IPSS) that includes a number of variables: age (older than 65 years), the presence of constitutional symptoms, hemoglobin <10 g/dL, WBC count >25×10^9^/L and the presence of blasts in the PB. These factors define four risk groups: low risk (no factors), intermediate risk-1 (one factor), intermediate risk-2 (two factors), high risk (three or more factors) [14]. An additional prognostic score has been considered, the dynamic IPSS (DIPSS) that utilizes the same prognostic variables used in IPSS but can be applied at any time during the course of the disease [15]. Spleen index is the product of the longitudinal by the transversal spleen axis, the latter defined as the maximal width of the organ. PB samples from 13 CTRLs comparable for age were studied. The study was approved by the local Ethic Committee.

### Genotypes and allele burden

All subjects had genetic analyses for driver mutations in blood granulocytes. *JAK2*V617F and *MPL*W515 mutations were detected by real-time quantitative PCR. High Resolution Melting analyses followed by bi-directional Sanger sequencing was also used to test for *MPL* mutations. *CALR* indels were identified by High Resolution Melting analysis. The assessment of A3669G polymorphism of the corticosteroid receptor was performed by High Resolution Melting analysis and confirmed by direct sequencing [16].

### Flow cytometry analysis

We measured constitutive and cytokine induced phosphorylation of STAT3, STAT5, and ERK1/2 in the PB CD34^+^ cells by a phospho-specific flow cytometry assay. The signaling activity was measured in 400 μl of EDTA anticoagulated PB cells before (to measure constitutive phosphorylation) and following incubation with recombinant IL6 (Miltenyi BiotecGmbH, Bergisch Gladbach, Germany), thrombopoietin (TPO-Peprotech, Rocky Hill, NJ, USA), or phorbol 12-myristate 13-acetate (PMA-Sigma Aldrich, St. Luis, MO, USA) for 15 minutes at 37°C, according with manufacturer’s instructions. Red cells were lysed and white cells fixed by adding pre-warmed 1X Lyse/Fix buffer (BD Biosciences, San Jose, CA, USA) for 15 minutes at 37°C. PB cells were washed in Phosphate Buffered Saline (PBS) and permeabylized by ice cold Perm buffer III (BD Biosciences) for 30 minutes at 4°C. Ten microliters of anti-human CD34-FITC (BD Biosciences) were added to all the conditions. Antibodies used for phosphoprotein detection (p-STAT3, p-STAT5, p-ERK1/2) were Alexa Fluor647-conjugated (20 μl) (BD Biosciences). Isotype controls were used at the same concentration as test antibodies. Incubation was performed at room temperature for 1 hour in the dark. The same staining procedure was adopted to assess the total STAT5 protein in CD34^+^ cells, by adding 10 μl of anti-human CD34-PE (BD Biosciences) and 10 μl of anti-human STAT5-FITC (Thermo Fisher Scientific, Rockford, IL, USA). PB cells (5x10^5^-1.5x10^6^) were acquired by a Navios flow cytometer (Beckman Coulter, Inc, Brea, CA), and analysed by Kaluza flow analysis software (Beckman Coulter). Based on their fluorescence, CD34^+^ cells were electronically gated. The median fluorescence intensity (MFI) values were obtained by dividing the MFI of the specific antibody by the MFI of the isotype control. “MFI” in the manuscript will refer to this ratio.

One hundred microliters of EDTA anticoagulated blood was stained with anti-human CD34-FITC and anti-human CXCR4-PE (BD Biosciences). After red cell lysis the samples were centrifuged and the pellets resuspended in 300 μL of saline. Results were expressed as percentage of CD34^+^ cells co-expressing CXCR4.

### Western blotting

Human CD34^+^ cells were lysed for 30 minutes at 4°C in HEPES-glycerol lysis buffer (50 mM Hepes, 150 mM NaCl, 10% glycerol, 1% Triton X-100, 1.5 mM MgCl_2_, 1 mM EGTA) containing 1 μg/mL leupeptin and 1 μg/mL aprotinin. Afterwards, samples were clarified by centrifugation at 15700*g* at 4°C for 15 min. Laemmli sample buffer was then added to supernatants. Samples were heated at 95°C for 3 minutes, separated by electrophoresis on 12% sodium dodecyl sulfate-polyacrylamide gel and then transferred to polyvinylidine fluoride membranes. Membranes were probed with primary antibodies, washed 3 times with PBS and Tween 0.1% and incubated with peroxidase-conjugate secondary antibodies. Membranes were visualized using Immobilon western chemiluminescent HRP substrate (Millipore, Burlington, MA, USA) and images were acquired by UVITEC Alliance Mini HD9 (Eppendorf, Hamburg, Germany), and the protein levels detected were quantified using UVITEC NineAlliance 1D software.

### Statistics

The groups were compared by means of Mann-Whitney U-test for unpaired samples or Wilcoxon for paired samples when indicated; ANOVA of Kruskall-Wallis and Bonferroni’s correction as a post hoc test were used for multiple comparison. Spearman correlation test was used when indicated. All computations were performed with STATISTICA software (StatSoft, Inc. Tulsa, OK, USA).

## Results

### Patients and controls

Two subjects (3.5%) had constitutive and cytokine induced analysis of STAT5, STAT3, and ERK1/2 signaling activity determined at diagnosis and 55 (96.5%) after diagnosis (median time from diagnosis 82.0 months, range, 1–389 months). Demographic, clinical, biological, and molecular variables of the analyzed population are displayed in Table 1. None of the patients was receiving therapy at time of sampling.

**Table 1:** Demographic, hematological and genetic characteristics of the study population with primary myelofibrosis at the time of peripheral blood sampling (n = 57).

	n/57	
**Male, number (%)**	31	(54.4%)
**Age (years), median (range)**	57	53.0 (31–76)
**BMI (kg/m**^**2**^**), median (range)**	37	22.8 (17.5–37.5)
**Time from diagnosis to examination (months), median (range)**	57	81.0 (0–389)
**Hemoglobin concentration (g/dL), median (range)**	57	12.6 (7.6–17.1)
**White-blood cell count (x10**^**9**^**/L), median (range)**	57	8.5 (2.7–45)
**Immature myeloid cells in blood (%), median (range)**	19	2.0 (1–15)
**Blasts in blood (%), median (range)**	20	1.0 (1–6)
**Monocytes in blood (x10**^**9**^**/L), median (range)**	57	549 (75–25560)
**Platelet count (x10**^**9**^**/L), median (range)**	57	439 (49–1243)
**Spleen index (cm**^**2**^**), median (range)**	57	120 (90–756)
**CD34**^**+**^ **cell in blood (x10**^**6**^**/L), median (range)**	57	26.0 (0.9–1849)
***JAK2* V617F mutation, number (%)**	34	(59.7%)
***JAK2* V617F mutation with <50% allele burden, number (%)**	22	(38.6%)
***JAK2* V617F mutation with ≥50% allele burden, number (%)**	12	(21.1%)
***CALR* mutation, number (%)**	19	(33.3%)
***CALR* 52 bp deletion (type 1), number (%)**	14	(24.6%)
***CALR* 5 bp insertion (type 2), number (%)**	5	(8.7%)
***MPL* W515 mutation, number (%)**	4	(7%)

### Measurement of constitutive and cytokine induced STAT3, STAT5, ERK1/2 phosphorylation by flow cytometry

The detection of intracellular signaling pathways by flow cytometry has been shown to be reproducible [9], and it was found to be of comparable sensitivity to Western blotting [17]. We performed this technique acquiring 5x10^5^-1.5x10^6^ PB cells, and evaluating the signaling activity in electronically gated CD34^+^ cells. We assessed the reproducibility of our assay testing 4 patients in 2 separate occasions (median months 4, range 1–24) during a stable phase of disease; the values obtained were comparable in both constitutive (p = 0.87) and cytokine induced condition (p = 0.79) (S1 Fig).

**Fig 1:**
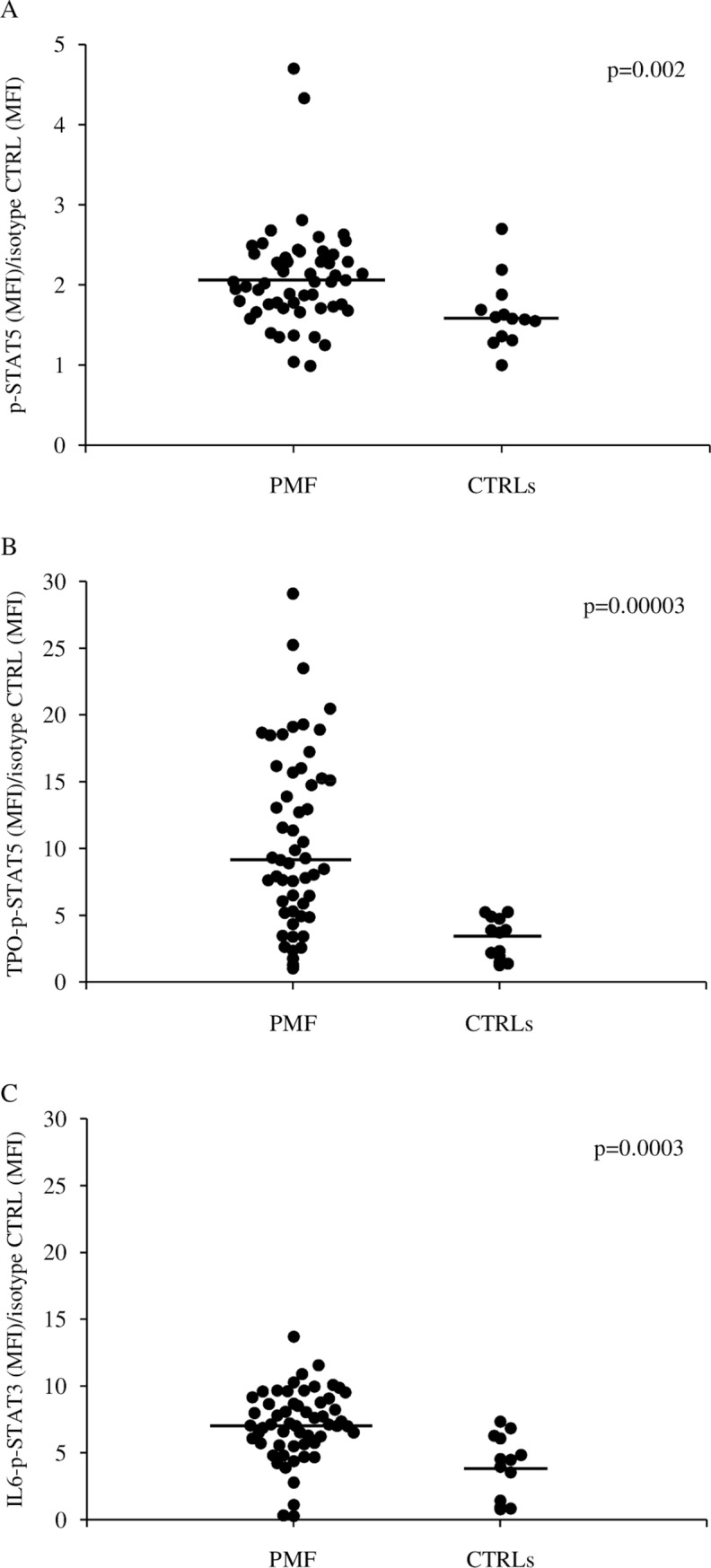
STAT3/STAT5 constitutive and cytokine induced phosphorylation in CD34^+^ cells. Evaluation of constitutive p-STAT5 (A), TPO induced p-STAT5 (B), and IL6 induced p-STAT3 (C) in circulating CD34^+^ cells of patients with primary myelofibrosis (PMF) and healthy subjects (CTRLs). Median values are shown as solid lines.

From now on, the acronym “MFI” refers to the ratio: MFI of specific antibody / MFI of the isotype control. The constitutive phosphorylation of STAT5 (p-STAT5) in circulating CD34^+^ cells was significantly higher (p = 0.002) in patients with PMF (n = 57) than in CTRLs (n = 13) (Fig 1A), while p-STAT3 (MFI 1.0, range 0.04–2.4) and p-ERK1/2 (MFI 1.5, range 0.3–3.1) were comparable to those of circulating CD34^+^ cells from CTRLs (p-STAT3 MFI 1.0, range 0.3–1.6; p-ERK1/2 MFI 1.4, range 1.0–1.9). The TPO induced p-STAT5 and IL6 induced p-STAT3 were significantly higher (p = 0.00003 and p = 0.0003, respectively) in CD34^+^ cells from patients with PMF than in CTRLs (Fig 1B and 1C, respectively), while PMA induced p-ERK1/2 was comparable in patients (MFI 1.57, range 1.1–3.6) and CTRLs (MFI 1.4, range 1.0–2.0). Similar results were obtained when patients with P-PMF (n = 20) with degree of fibrosis (0–1) were separately compared to CTRLs (p-STAT5: p = 0.02, TPO induced p-STAT5: p = 0.0006, IL6 induced p-STAT3: p = 0.007).

We analyzed the determinants of elevated constitutive and cytokine induced p-STAT5 and the IL6 induced p-STAT3 by correlating their MFI values with parameters whose causal role on signaling activation was plausible. We found no significant correlations with age, sex, body mass index, or presence/absence of the A3669G polymorphism of the corticosteroid receptor (S2–S5 Figs).

**Fig 2:**
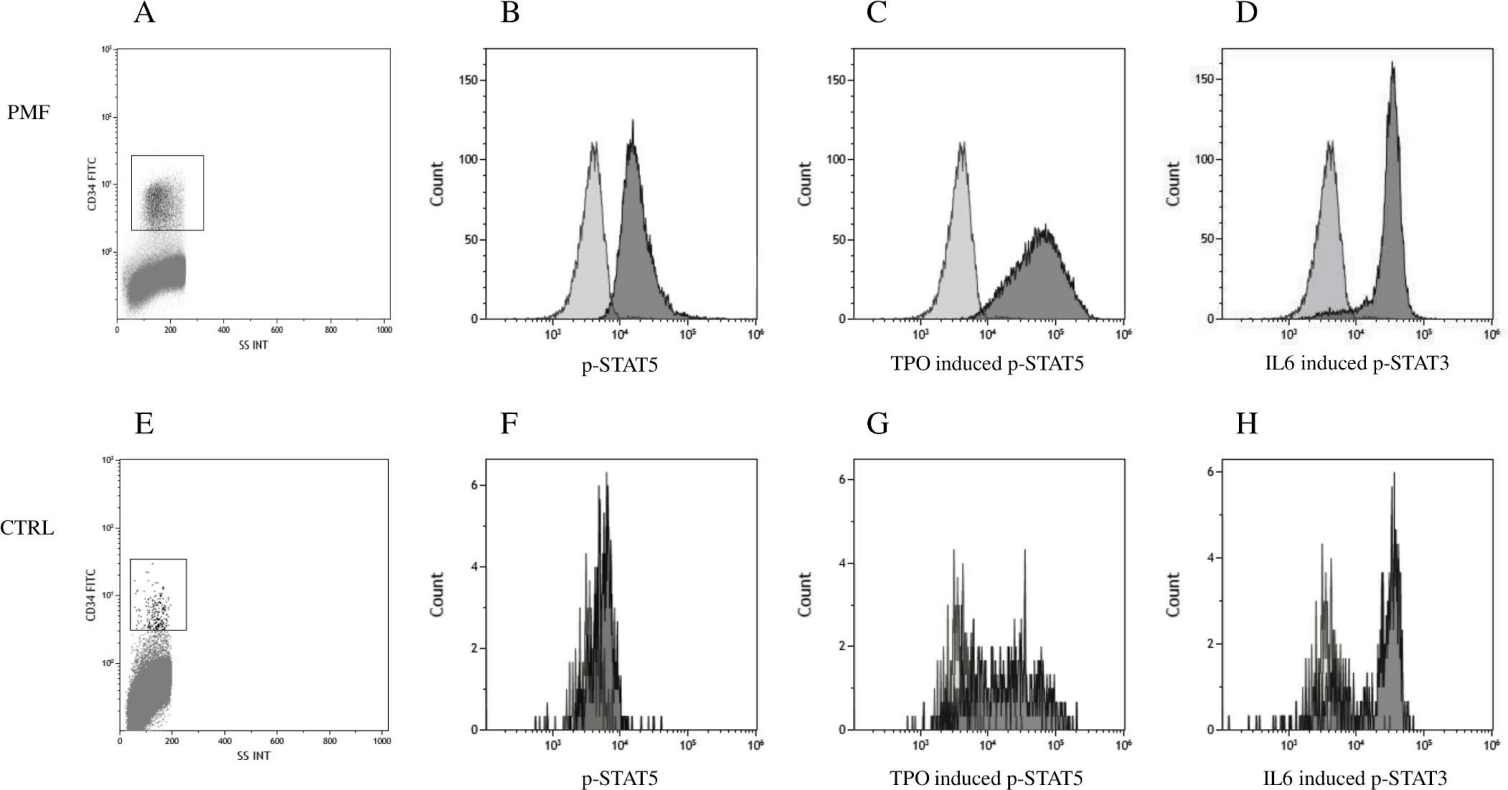
STAT3/STAT5 constitutive and cytokine induced phosphorylation analysis by flow cytometry. Cytofluorimetric plots of CD34^+^ cell gating in a representative patient with primary myelofibrosis (PMF) (A) and a healthy subject (CTRL) (E). Overlay histograms show the isotype control (light grey) and constitutive p-STAT5 (B and F), TPO induced p-STAT5 (C and G) and IL6 induced p-STAT3 (D and H) signals.

Cytofluorimetric plots for CD34^+^ cell gating and histograms for constitutive p-STAT5, TPO induced p-STAT5 and IL6 induced p-STAT3 from a representative patient with PMF (Fig 2A–2D) and a representative CTRL (Fig 2E–2H) are shown.

**Fig 3:**
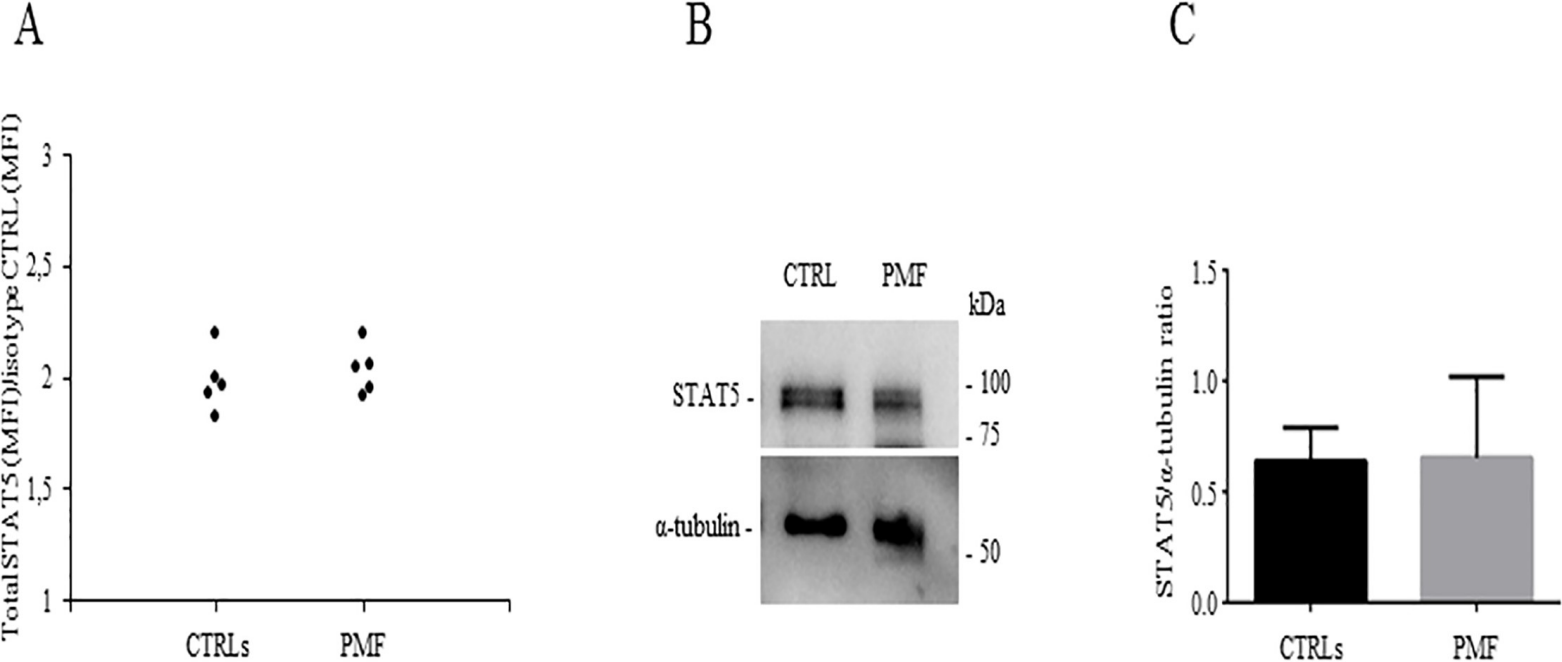
STAT5 evaluation. Assessment of STAT5 by flow cytometry (A) in CD34^+^ cells from patients with primary myelofibrosis (PMF) (n = 5) and healthy subjects (CTRLs) (n = 5). Western blot analysis of STAT5 in CD34^+^ cells derived from CTRLs and patients with primary myelofibrosis (PMF); membranes were probed with anti-STAT5 antibody (CST 94205) and with anti-α-tubulin antibody (Abcam ab52866) as loading control (B for one representative patient and CTRL). Densitometric analysis of STAT5/α-tubulin ratio (n = 5 CTRLs, n = 5 PMF) (C).

To investigate the total STAT5 expression, we stained PB cells from patients (n = 5) and CTRLs (n = 5) with a PE-conjugated anti-human CD34 and a FITC-conjugated anti-human total STAT5 antibodies. These experiments showed that the MFI values of total STAT5 were comparable in patients and CTRLs (Fig 3A). This finding was confirmed by Western blot analysis (Fig 3B and 3C). These data indicate that the increase of p-STAT5 MFI values in patients with PMF are not related to differences in the total STAT5 protein expression.

### Driver mutations and p-STAT5 signaling of circulating CD34^+^ cells from patients with PMF

**Fig 4:**
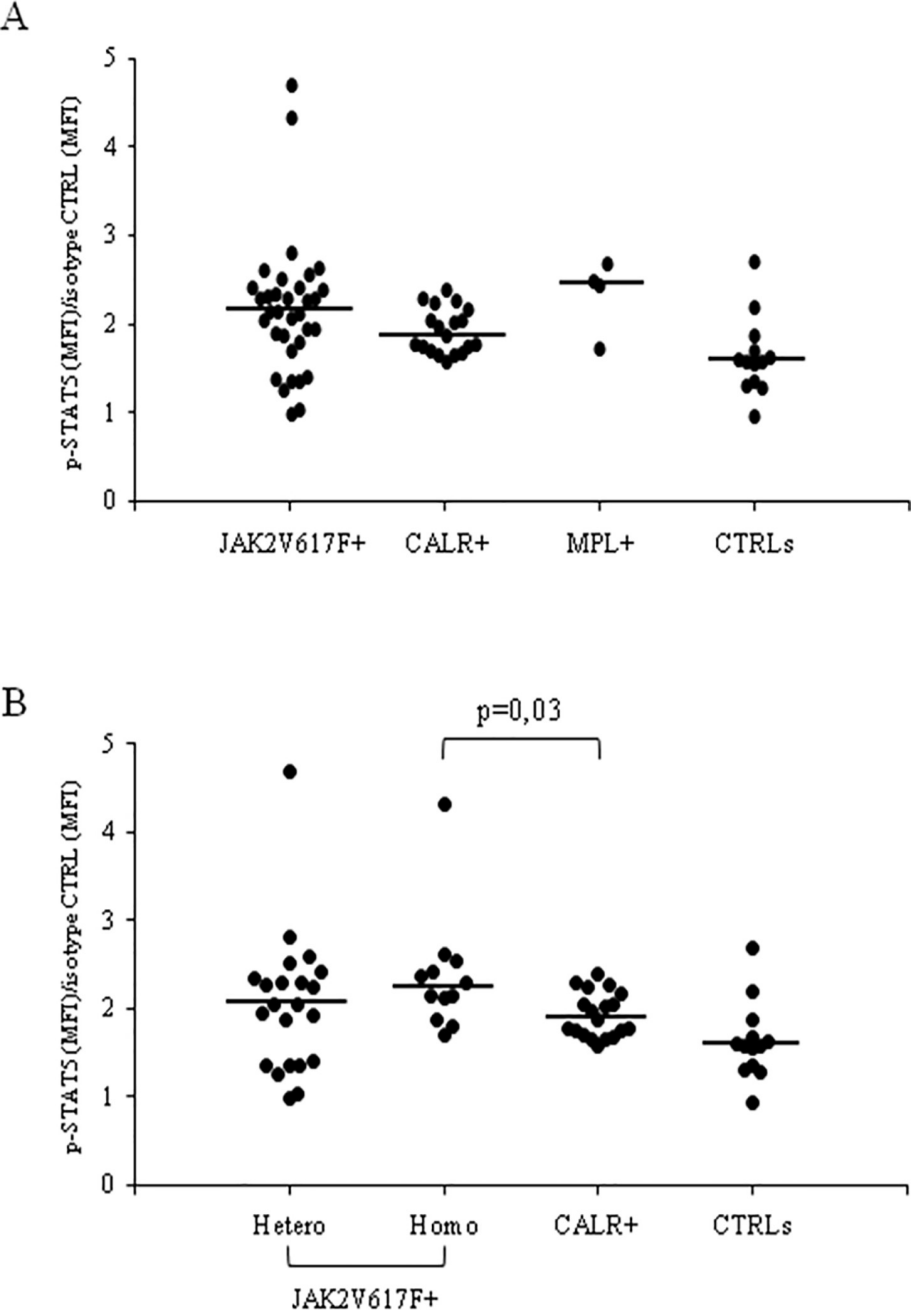
Constitutive STAT5 phosphorylation and disease genotypes. Constitutive p-STAT5 signal in circulating CD34^+^ cells of healthy subjects (CTRLs) and of patients with primary myelofibrosis (PMF) divided according with the genotype (A) and according with the genotype and the allele burden within the *JAK2*^+^ patients (B). Median values are shown as solid lines.

When patients were divided according with their genotype and compared to CTRLs (ANOVA of Kruskall-Wallis p = 0.005), the constitutive p-STAT5 MFI values in circulating CD34^+^ cells from *JAK2*V617F^+^ (n = 34) and *CALR*^+^ (n = 19) patients with PMF were higher than those of CTRLs (p = 0.027 and p = 0.01, respectively); in *MPL*^+^ patients (n = 4) the p-STAT5 MFI values, possibly due to the limited number, were increased but not significantly (p = 0.06) higher than CTRLs (Fig 4A).

In addition within the *JAK2* mutant patients, the homozygous ones had CD34^+^ p-STAT5 MFI values higher than those of CTRLs and of patients with *CALR*^+^ genotype (p = 0.003 and p = 0.03, respectively), while the heterozygous had p-STAT5 MFI values comparable to those found in CD34^+^ cells of CTRLs and of *CALR*^+^ patients (ANOVA of Kruskall-Wallis p = 0.005) (Fig 4B).

**Fig 5:**
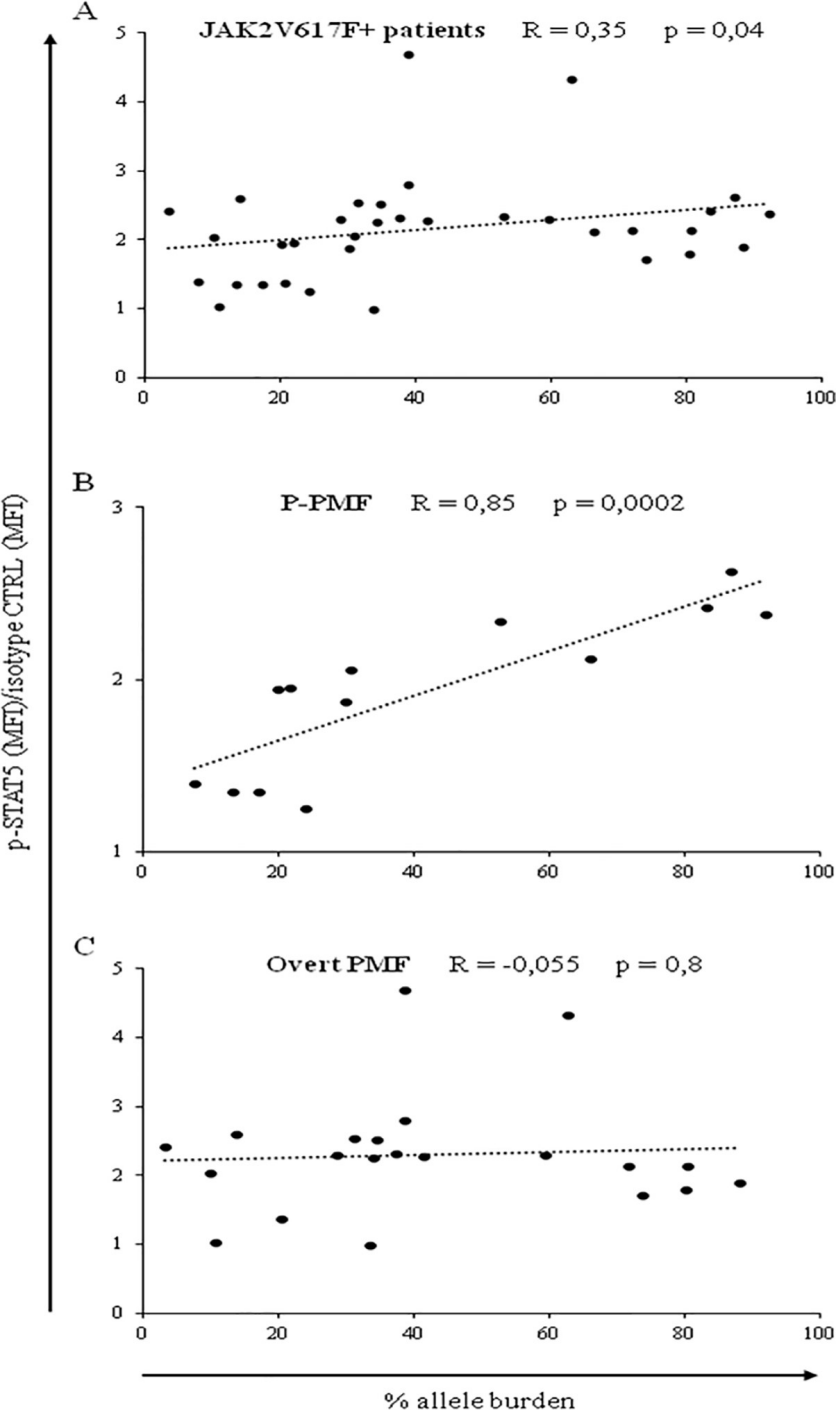
Constitutive STAT5 phosphorylation correlates with *JAK*2V617 allele burden. Significant direct correlation between constitutive p-STAT5 MFI values in PB CD34^+^ cells and *JAK2* mutated allele burden in patients with PMF (A). The same correlation is shown in patients with prefibrotic-PMF (P-PMF) (B) and overt primary myelofibrosis (PMF) (C).

Further investigating the *JAK2* mutant patients, we evidenced a significant (R = 0.35, p = 0.04) direct correlation between constitutive p-STAT5 MFI values and the mutant allele burden (Fig 5A). We found that the significant correlation between constitutive p-STAT5 MFI values in CD34^+^ cells and the mutant allele burden was present in patients with P-PMF (n = 13, R = 0.85, p = 0.0002), but not in patients with overt PMF (n = 21, R = -0.055, p = 0.8) (Fig 5B and 5C, respectively).

No significant correlation was found between the genotype or allele burden and the TPO induced p-STAT5 or IL6 induced p-STAT3 MFI values in CD34^+^ cells (S6 and S7 Figs). No difference was found in the signaling pathways tested in circulating CD34^+^ cells between *CALR*^+^ patients expressing the 52-bp deletion (type 1) (n = 14) or the 5-bp insertion (type 2 mutation) (n = 5), as the most frequent variants (S8 Fig).

### Anemia, CD34^+^ cell number, and CD34^+^CXCR4^+^ cell frequency are associated with altered constitutive p-STAT5 or IL6 induced p-STAT3 signaling pathways in patients with PMF

In patients with PMF the CD34^+^ cell constitutive p-STAT5 MFI values were significantly inversely correlated with hemoglobin levels (R = -0.28, p = 0.037), one of the most relevant hematological and clinical variable, whose variations portrait disease progression; no correlation was found with IPSS, DIPSS, WBC, monocyte, or platelet count, frequency of circulating blood blasts, serum LDH, spleen size and degree of BM fibrosis (S1 Table).

Among the major biological parameters of disease progression, constitutive p-STAT5 MFI values were significantly inversely correlated with both the frequency of CD34^+^CXCR4^+^ cells (R = -0.47 p = 0.0007), whose reduction marks the increase of the disease severity [18], and the serum cholesterol concentration (R = -0.51, p = 0.009); in addition, constitutive p-STAT5 MFI values were significantly directly correlated (R = 0.38, p = 0.005) with the number/L of circulating CD34^+^ cells.

IL6 induced p-STAT3 MFI values of CD34^+^ cells from patients with PMF were significantly directly correlated with the CD34^+^ number/L (R = 0.36, p = 0.008), and significantly inversely correlated with the frequency of CD34^+^CXCR4^+^ cells (R = -0.31, p = 0.027). No significant correlation was found when we considered the MFI values of TPO induced p-STAT5 (S1 Table).

Constitutive p-STAT5 or IL6 induced p-STAT3 MFI values and markers of disease progression were separately performed in the *JAK2*V617F and *CALR* mutants (n = 34 and n = 19, respectively).

**Fig 6:**
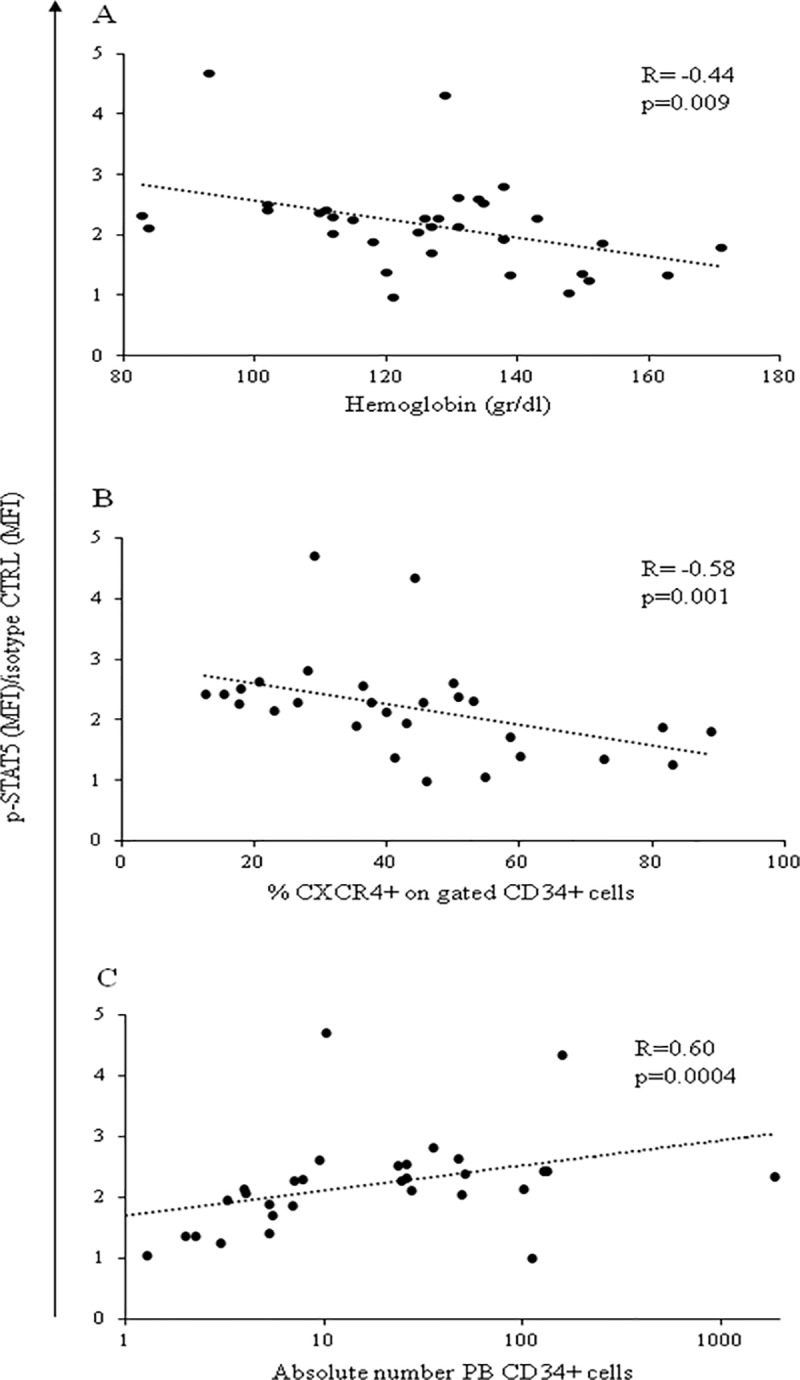
Correlations between constitutive p-STAT5 MFI values and disease parameters. Significant direct correlation between constitutive p-STAT5 MFI values in PB CD34^+^ cells and hemoglobin levels (A), CD34^+^ expressing CXCR4 frequency (B), and the absolute number of circulating CD34^+^ cells (C) in *JAK2*^+^ patients with PMF.

The correlations described in the whole population of patients with PMF characterized the *JAK2*V617F^+^ patients (Fig 6A–6C). In fact, as shown in Table 2, in the *CALR* mutant subjects we evidenced only a significant inverse correlation of constitutive p-STAT5 MFI values with the cholesterol serum levels (R = -0.97, p = 0.0004).

**Table 2:** Correlations between constitutive p-STAT5, TPO induced p-STAT5, and IL6 induced p-STAT3 MFI values and disease parameters tested in circulating CD34^+^ cells of *CALR*^+^ patients with PMF.

		Hb	CD34^+^CXCR4^+^frequency	Chol	CD34^+^ cells x10^6^L
**Constitutive p-STAT5**	R	0.08	-0.2	**-0.97**	0.2
	p	0.7	0.5	**0.0004**	0.5
**TPO induced p-STAT5**	R	0.4	-0.26	-0.46	0.03
	p	0.11	0.35	0.29	0.9
**IL6 induced p-STAT3**	R	0.09	-0.3	-0.4	0.24
	p	0.7	0.26	0.34	0.35

Hemoglobin: Hb Cholesterol: Chol

Moreover, among the *JAK2*V617^+^ patients, those with P-PMF determined the significant correlation of constitutive CD34^+^ cell p-STAT5 MFI values with the hemoglobin levels and the PB CD34^+^CXCR4^+^ cell frequency (Table 3). At variance, a significant correlation of constitutive p-STAT5 MFI values with the absolute number of circulating CD34^+^ cells was present in *JAK2*V617^+^ patients either with P-PMF or PMF (Table 3). Only patients with overt PMF showed a correlation of TPO-induced p-STAT5 and IL6 induced p-STAT3 MFI values with the absolute number of circulating CD34^+^ cells (S2 Table).

**Table 3:** Correlations between CD34^+^ cell constitutive p-STAT5 MFI values and disease progression markers in patients with prefibrotic-PMF (P-PMF) or overt PMF.

	*JAK2*V617F^+^Patients		Hb	CD34^+^CXCR4^+^frequency	CD34^+^ cells x10^6^L
**Constitutivep-STAT5**	P-PMF	R	**-0.72**	**-0.88**	**0.85**
	n = 13	p	**0.005**	**0.0016**	**0.001**
**Constitutivep-STAT5**	PMF	R	-0.27	-0.37	**0.50**
	n = 21	p	0.24	0.1	**0.027**

Hemoglobin: Hb Cholesterol: Chol

## Discussion

In this study, we have investigated the constitutive and cytokine induced p-STAT3, p-STAT5 and p-ERK1/2 MFI values in circulating CD34^+^ cells of patients with PMF, taking advantage of a cytofluorimetric method. This approach allowed us to achieve a number of goals; first, acquiring up to 1,5x10^6^ PB cells we are able to analyse very low number of circulating CD34^+^ cells in both CTRLs and patients. Second, by electronically gating the CD34^+^ cells during the analysis, we avoided immune-magnetic bead cell selection and the possible mechanical activation of them during the procedure. Third, we avoided the necessity of great quantity of blood necessary to quantify STAT phosphorylation in CD34^+^ cells by Western blotting, allowing the detection also in anemic patients.

We demonstrate that CD34^+^ cells of patients with PMF have higher constitutive p-STAT5, and cytokine induced p-STAT5 and p-STAT3 MFI values than those of CTRLs. We suggest that the increase of constitutive p-STAT5 is actual, since the presence of total STAT5 protein is comparable in patients and CTRLs by both flow cytometry and Western blotting. The pattern of phosphorylation does not completely reflect what has been reported in PB CD34^+^ cells and granulocytes of patients with PMF, where constitutive p-STAT3, but not constitutive p-STAT5, has been found increased compared to CTRLs [10, 19–21]. In addition, Anand et al [9] described higher values of constitutive p-ERK1/2 in CD34^+^ cells from BM of MF patients, while Teofili et al, using an immune-histochemical approach, did not find any difference in both constitutive p-STAT5 and p-STAT3 in BM CD34^+^ cells of patients with MF compared to subjects without a myeloproliferative disease [8]. An explanation of the difference between our results and published data is not obvious; however, the detection method, the cell type analysis, and the disease phenotype could be responsible for the discrepancies. More importantly, constitutive p-STAT3 levels have been associated in MPNs to an increase of inflammatory cytokine secretion rather than to a proliferative phenotype [22, 23]. The lack of increase of constitutive p-STAT3 MFI values may reflect a low level of inflammatory component in our cohort of patients, who showed plasma high sensitive C reactive protein levels (mean 0.3mg/dL, standard deviation 0.3) well below the values (mean 0.6 mg/dL, standard deviation 1.3) that we observed in a larger cohort (n = 526) of patients with PMF [24]. Anyhow, the significantly increased values of IL6 induced p-STAT3 indicate that this pathway is more prone to activation in patients with PMF, even in a stable phase of the disease, than in CTRLs.

A second, original finding of our work is that constitutive p-STAT5 MFI values differ according to the genotype, with homozygous *JAK2*- mutated CD34^+^ cells showing higher constitutive p-STAT5 MFI values than the *CALR*^+^ ones. It is possible that the different molecular mechanism of activation of the JAK/STAT pathway in *CALR*- mutated cells compared to *JAK2* homozygous- mutation explains this quantitative difference. Similar differences were found between *JAK2*- mutated and *CALR*- mutated patients with MPN affected by ruxolitinib withdrawal syndrome [25]. We also found that the allele burden of *JAK2*V617F- mutated CD34^+^ cells directly correlates with the constitutive p-STAT5 MFI values of patients with PMF at variance with a previous report performed in a limited number of patients [9]. Corroborating our observation, the granulocyte gene expression profiling obtained from patients with MPN showed that the JAK/STAT pathway activation signature was strikingly higher in patients with a high *JAK2*V617F allele burden [10]. It is difficult to understand why the correlation between *JAK2*V617F- mutated CD34^+^ cells and the constitutive p-STAT5 MFI values becomes stronger within patients with P-PMF; however, since it has been reported that the allele burden is correlated with the disease severity in both *JAK2*- mutated patients with PMF [24], and in *JAK2*- mutated animal models [26], the correlation of the *JAK2*- mutated allele burden with constitutive p-STAT5 MFI values support the concept that the phosphorylation of this transcription factor has a role in fueling the disease progression.

It has to be considered that we have measured constitutive p-STAT5 values in CD34^+^ cells, while we have correlated them with the *JAK2*V617F allele burden assessed in granulocytes. An unbalanced amplification of the *JAK2*- mutated versus non-mutated hematopoiesis in the PB could underestimate or bias the correlation [9]; however, available data indicate that in MF, at variance with PV, the average mutant allele burden measured in the hematopoietic progenitor/stem cells remains stable during differentiation and is comparable to that of mature hematopoietic cells [27]. As a third novel finding of our research, we report that constitutive p-STAT5 activation inversely correlates with hemoglobin levels in patients with PMF. However, when this correlation was investigated considering patients with P-PMF (fibrosis 0–1 according WHO 2016) vs patients with overt PMF (fibrosis 2–3) the inverse correlation with hemoglobin was evident only for prefibrotic patients. Similarly, we observed that constitutive p-STAT5 MFI values inversely correlated with the frequency of circulating CD34^+^ cells expressing CXCR4 in patients with P-PMF but not in those with overt PMF. A potential interpretation of these observations is that in the prefibrotic disease the capacity of p-STAT5 of determining the phenotype is more pronounced than in the overt form, where it is likely that other pathogenetic determinants can confound the role and the effect of this transcription factor on the disease phenotype. As a matter of fact, these data, despite their interpretation, brings evidence to the concept that prefibrotic and overt PMF are distinct biological entity [13]. In physiological conditions, the binding of erythropoietin to its receptor activates the JAK/STAT5 pathway, resulting in the proliferation/amplification of the erythroid compartment. However, in PMF it is not unfrequent that patients present with normal or reduced hemoglobin levels, suggesting that in the stem/progenitor cell compartment, despite the p-STAT activation, erythroid differentiation is affected and does not depend only on the EpoR/JAK2/STAT5 pathway activation. This is consistent with the observation that in mouse models the absence of STAT5 does not affect the response to Epo [28]. It is therefore possible that in PMF hematopoiesis, at the stem/progenitor cell level, constitutively increased p-STAT5 induces the activation of genes that specifically counteract the EpoR pathway [29]; in particular, Interferon (IFN)-γ has been shown to be a pro-inflammatory protein that signals through the JAK/STAT pathway by binding of activated STAT5 to different target genes [30, 31], such as those of the apoptotic pathway, that could be responsible of the reduction of hemoglobin levels [32, 33].

Finally, we found that constitutive p-STAT5 MFI values were directly correlated with the frequency of circulating CD34^+^ cells, but inversely correlated with the CXCR4 expression on their membrane. Abnormal CD34^+^ cell mobilization is a well known specific biological feature of patients with PMF [1]. We have also previously shown that in patients with MF a reduced number of CD34^+^CXCR4^+^ circulating cells correlates with disease severity [18]. CXCR4 signaling, which occurs following the binding with its ligand SDF-1α/CXCL12, is known to involve p-STAT5 [34], thus the constitutive activation of p-STAT5 could result in a reduced expression of the CXCR4 on the cell membrane representing a potential mechanism of CD34^+^ cell mobilization from the BM into the PB. However, this cannot be the unique factor affecting CD34^+^ mobilization, since constitutive p-STAT5 is also active in progenitor cells of PV and ET patients [8], in whom this phenomenon is not detectable, at least in the chronic phase of the disease. To be noted, another transcription factor, nuclear factor erythroid 2-related factor 2 (Nrf2) has already been reported to have a direct role on the HPC CXCR4 expression [35] and has been shown to be involved in the regulation of CD34^+^ cell retention and homing to the BM in MF, characterized by the *JAK2*V617F-induced oxidative stress [36].

In summary, we have shown here that constitutive activation of the JAK/STAT pathway in circulating CD34^+^ cells of patients with PMF results in the increase of constitutive p-STAT5. We have also shown that p-STAT5 values are directly correlated with *JAK2*V617F allele burden and mark disease severity, both in term of hemoglobin reduction and of CD34^+^ cell mobilization in patients with P-PMF. Our data point toward a complex activation of STAT5-dependent pathways in the stem/progenitor compartment that characterizes PMF and its phenotypic diversity, and that will be matter of future investigations.

## Supporting information

S1 Fig Assessment of the reproducibility of STAT-ERK1/2 phosphorylation by flow cytometry. Four patients with primary myelofibrosis (PMF) were tested in 2 separate occasions (grey and black columns), during a stable phase of disease, for the constitutive p-STAT5, p-STAT3, and p-ERK1/2 signaling (A, B, C) and the TPO induced p-STAT5 (D), IL6 induced p-STAT3 (E) and PMA induced p-ERK1/2 (F) signaling in circulating CD34^+^ cells.
(TIF)

S2 Fig Correlation between age and constitutive p-STAT5 (A), TPO induced p-STAT5 (B), or IL6 induced p-STAT3 (C) MFI values in circulating CD34^+^ cells.
(TIF)

S3 Fig Correlation between sex and constitutive p-STAT5 (A), TPO induced p-STAT5 (B), or IL6 induced p-STAT3 (C) MFI values in PB CD34^+^ cells of patients with PMF.
(TIF)

S4 Fig Correlation between body mass index (BMI) and constitutive p-STAT5 (A), TPO induced p-STAT5 (B), or IL6 induced p-STAT3 (C) MFI values in PB CD34^+^ cells of patients with PMF.
(TIF)

S5 Fig Correlation between A3669G polymorphism of the corticosteroid receptor and constitutive p-STAT5 (A), TPO induced p-STAT5 (B), or IL6 induced p-STAT3 (C) MFI values in PB CD34^+^ cells of patients with PMF.
(TIF)

S6 Fig TPO induced p-STAT5 (A) and IL6 induced p-STAT3 (B) MFI values in PB CD34^+^ cells of patients with PMF divided according with the genotype. Median fluorescence intensity (MFI) median values of patients with different genotype (solid lines) and of healthy subjects (dotted line) are shown.
(TIF)

S7 Fig Correlation between *JAK2*V617F allele burden and TPO induced p-STAT5 (A), or IL6 induced p-STAT3 (B) MFI values in PB CD34^+^ cells of patients with PMF.
(TIF)

S8 Fig Constitutive p-STAT5 (A), TPO induced p-STAT5 (B) or IL6 induced p-STAT3 (C) MFI values in PB CD34^+^ cells of *CALR*^+^ patients expressing the 52-bp deletion (type 1 mutation) or the 5-bp insertion (type 2 mutation).
(TIF)

S1 Table Correlations between p-STAT5, TPO induced p-STAT5, and IL6 induced p-STAT3 pathways tested in circulating CD34^+^ cells of patients with PMF and disease parameters. (DOCX)

S2 Table Correlations between TPO induced p-STAT5, and IL6 induced p-STAT3 values tested in circulating CD34^+^ cells of *JAK2*V617F^+^ patients with PMF and disease parameters. (DOCX)
