# Supplementary figures and images for: Constitutive STAT5 phosphorylation in CD34+ cells of patients with primary myelofibrosis: Correlation with driver mutation status and disease severity
Source: PLoS One. 2019 Aug 1;14(8):e0220189. doi: 10.1371/journal.pone.0220189 (PMC6675063; doi:10.1371/journal.pone.0220189)

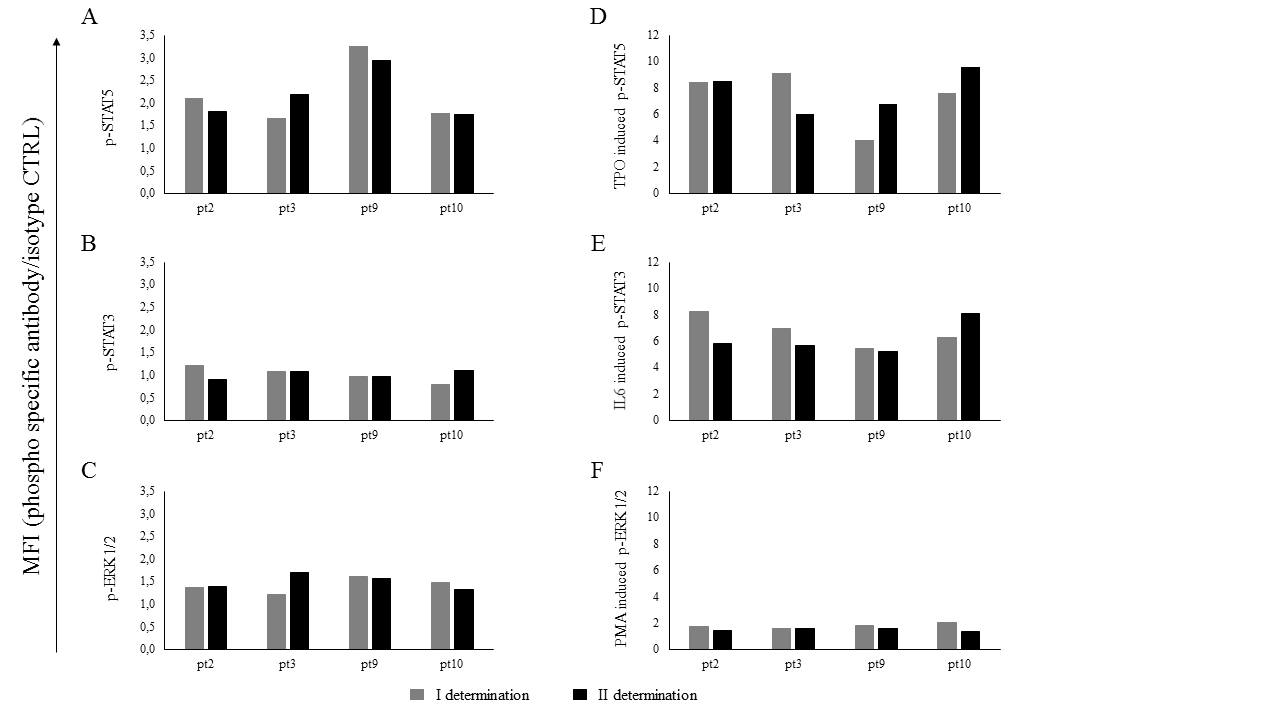

Supplement: S1 Fig — Four patients with primary myelofibrosis (PMF) were tested in 2 separate occasions (grey and black columns), during a stable phase of disease, for the constitutive p-STAT5, p-STAT3, and p-ERK1/2 signaling (A, B, C) and the TPO induced p-STAT5 (D), IL6 induced p-STAT3 (E) and PMA induced p-ERK1/2 (F) signaling in circulating CD34+ cells. (TIF) [file pone.0220189.s001.tif]

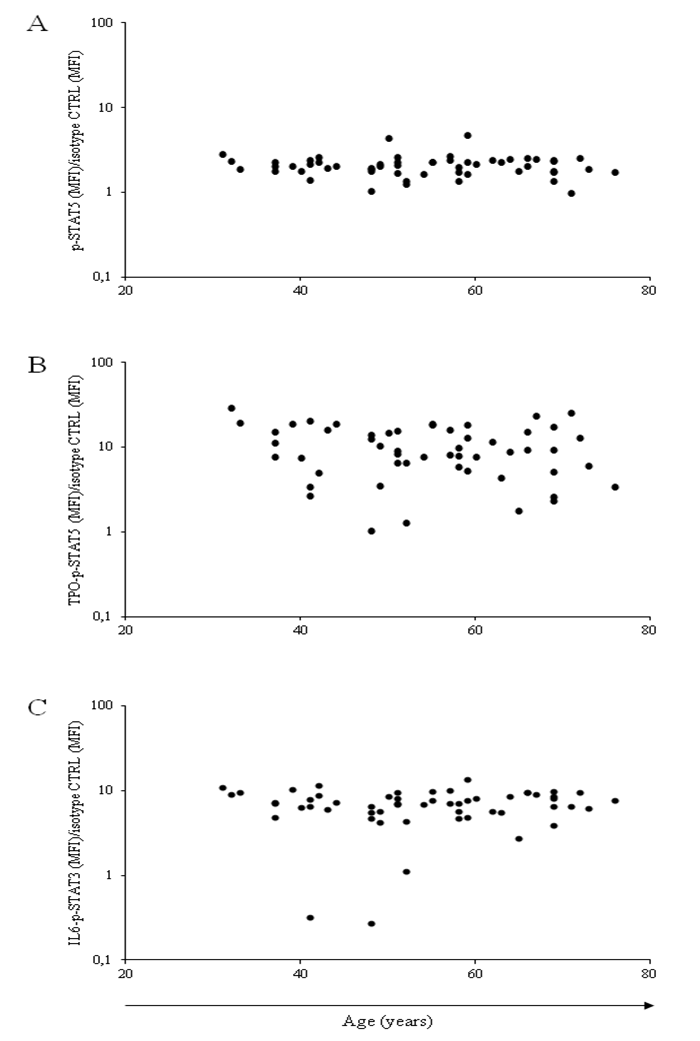

Supplement: S2 Fig — Correlation between age and constitutive p-STAT5 (A), TPO induced p-STAT5 (B), or IL6 induced p-STAT3 (C) MFI values in circulating CD34+ cells. (TIF) [file pone.0220189.s002.tif]

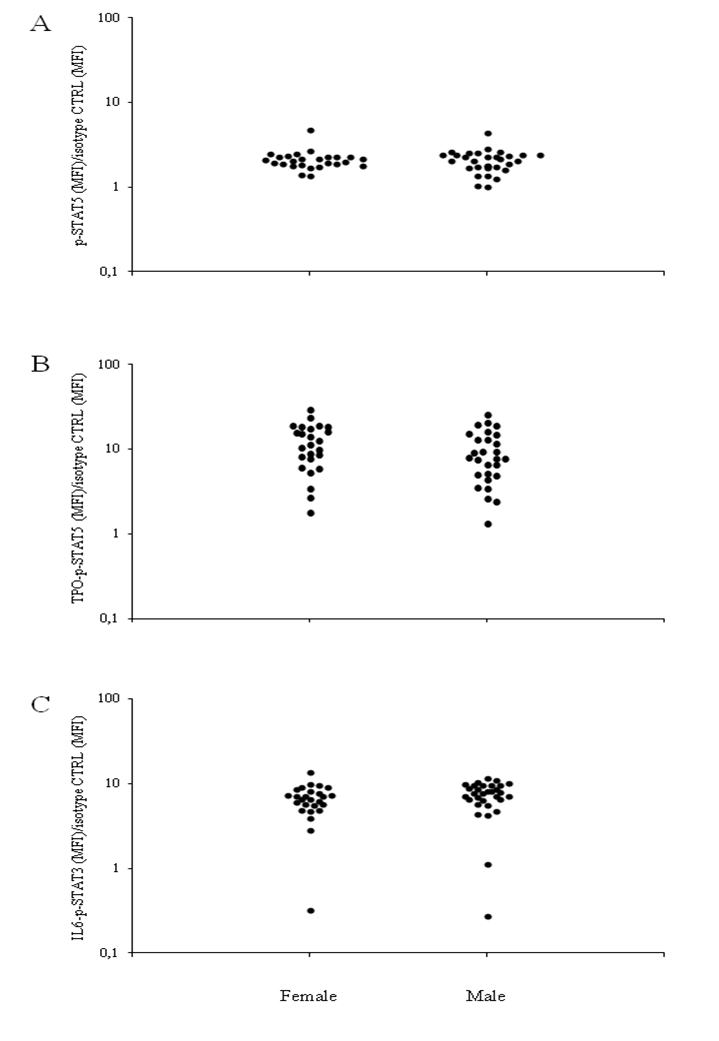

Supplement: S3 Fig — Correlation between sex and constitutive p-STAT5 (A), TPO induced p-STAT5 (B), or IL6 induced p-STAT3 (C) MFI values in PB CD34+ cells of patients with PMF. (TIF) [file pone.0220189.s003.tif]

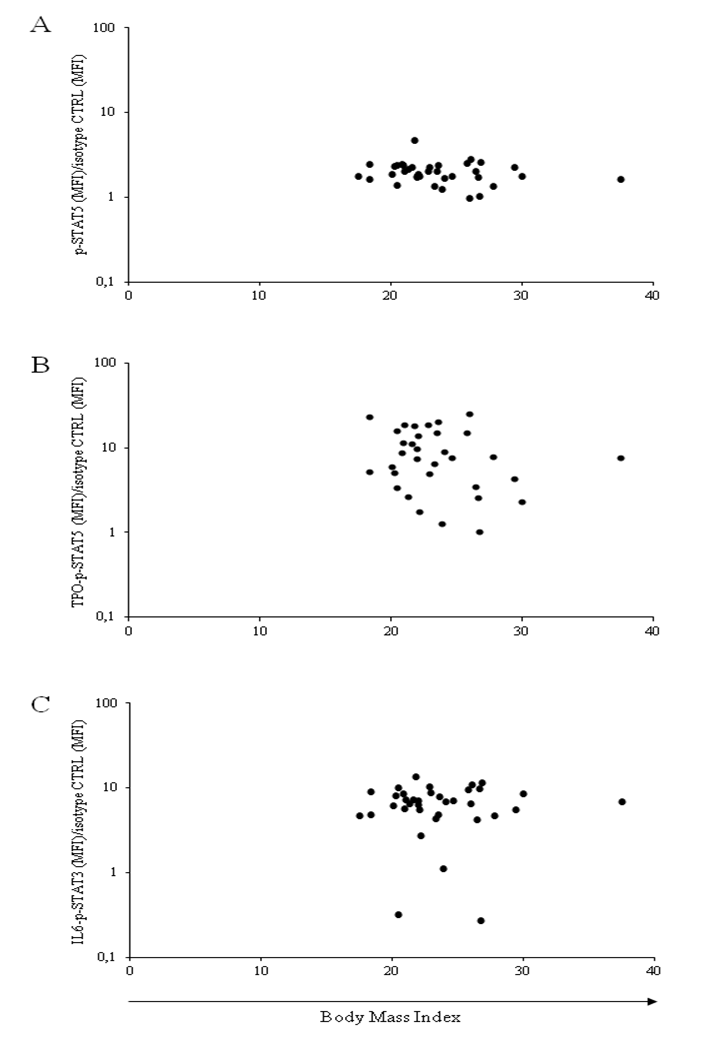

Supplement: S4 Fig — Correlation between body mass index (BMI) and constitutive p-STAT5 (A), TPO induced p-STAT5 (B), or IL6 induced p-STAT3 (C) MFI values in PB CD34+ cells of patients with PMF. (TIF) [file pone.0220189.s004.tif]

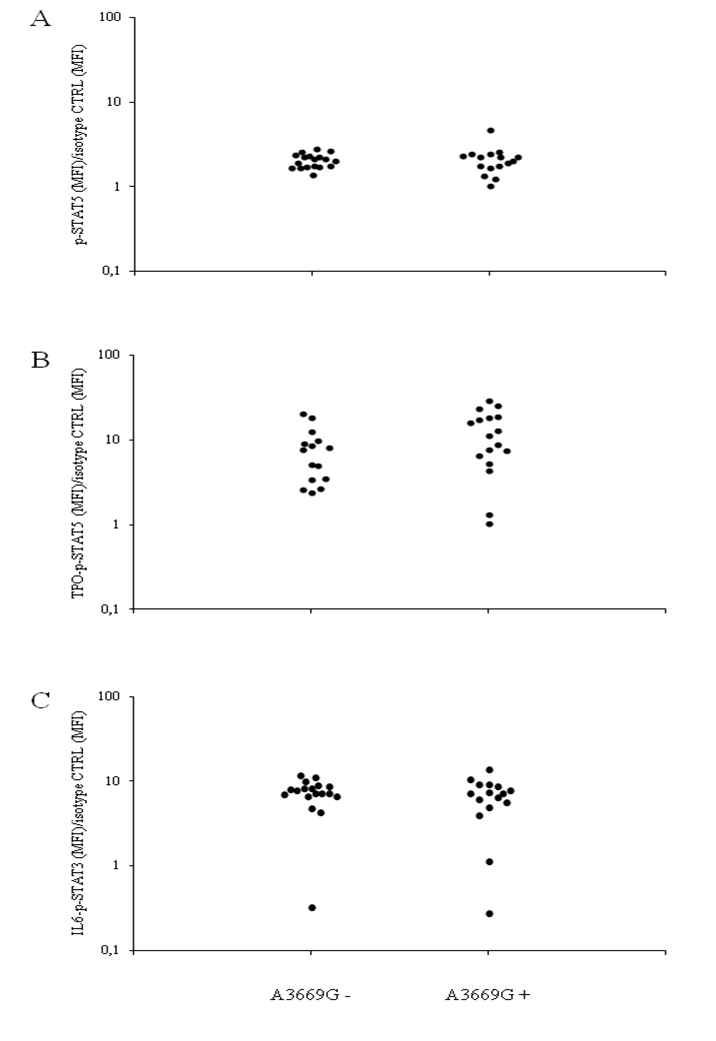

Supplement: S5 Fig — Correlation between A3669G polymorphism of the corticosteroid receptor and constitutive p-STAT5 (A), TPO induced p-STAT5 (B), or IL6 induced p-STAT3 (C) MFI values in PB CD34+ cells of patients with PMF. (TIF) [file pone.0220189.s005.tif]

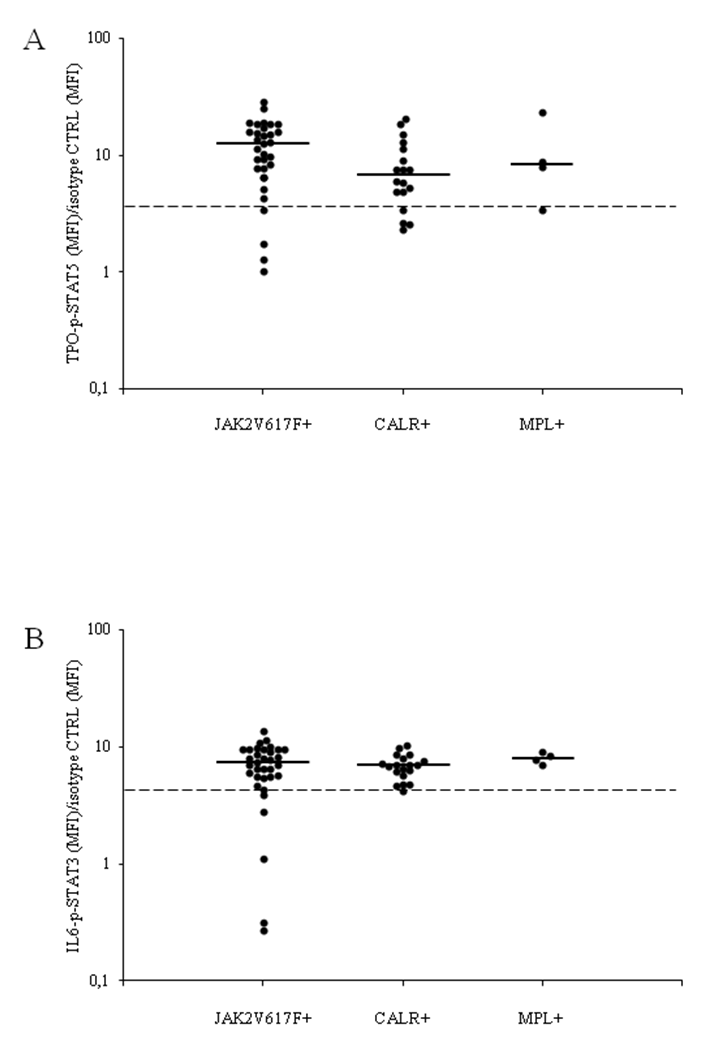

Supplement: S6 Fig — TPO induced p-STAT5 (A) and IL6 induced p-STAT3 (B) MFI values in PB CD34+ cells of patients with PMF divided according with the genotype. Median fluorescence intensity (MFI) median values of patients with different genotype (solid lines) and of healthy subjects (dotted line) are shown. (TIF) [file pone.0220189.s006.tif]

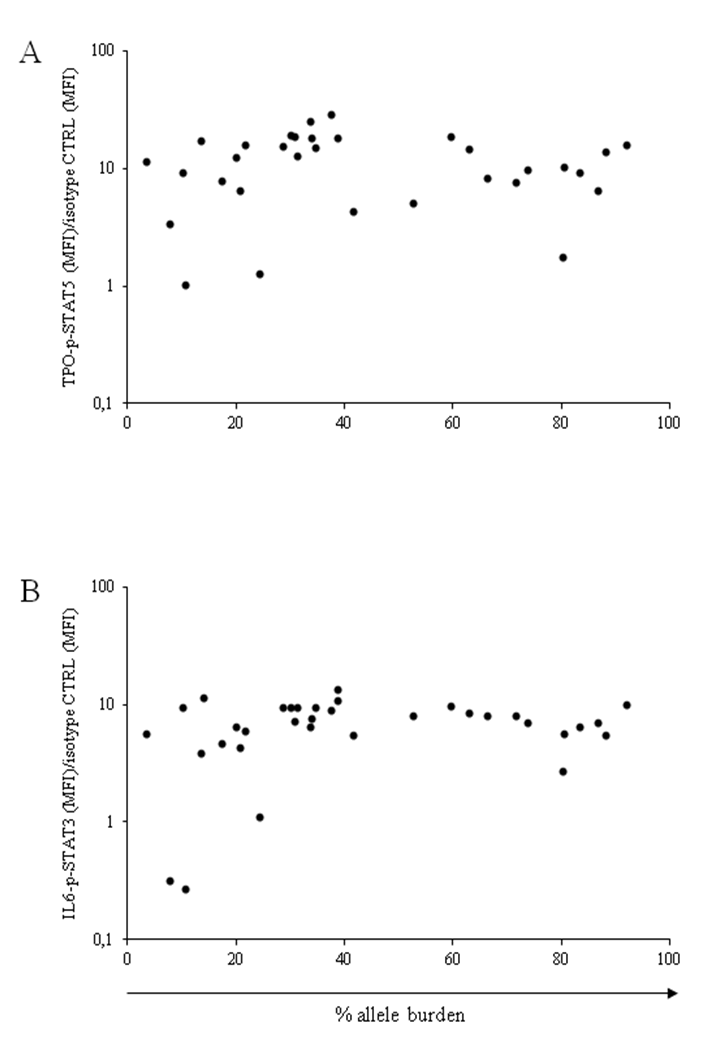

Supplement: S7 Fig — Correlation between JAK2V617F allele burden and TPO induced p-STAT5 (A), or IL6 induced p-STAT3 (B) MFI values in PB CD34+ cells of patients with PMF. (TIF) [file pone.0220189.s007.tif]

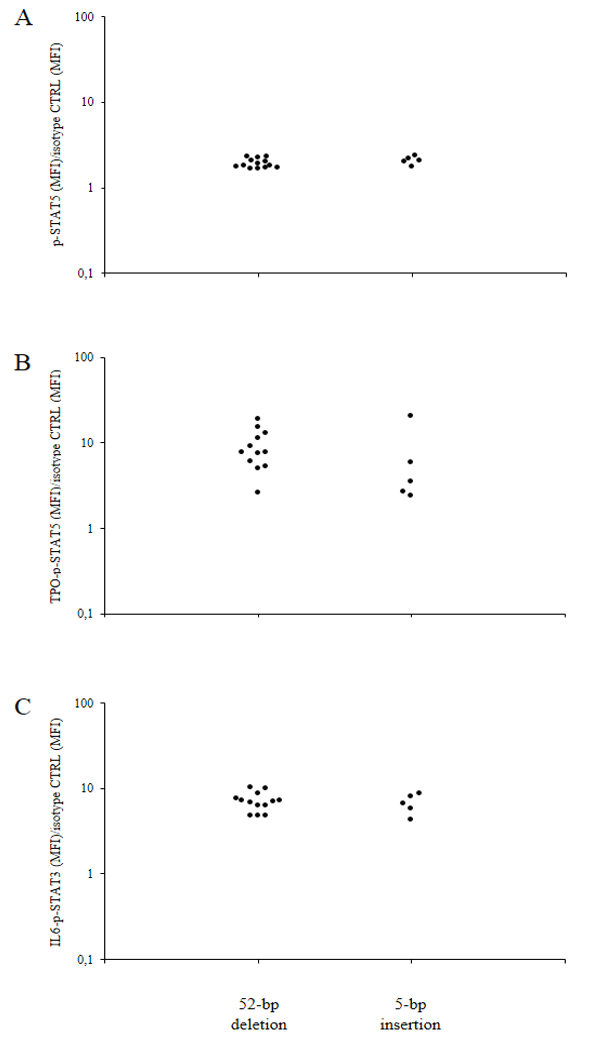

Supplement: S8 Fig — Constitutive p-STAT5 (A), TPO induced p-STAT5 (B) or IL6 induced p-STAT3 (C) MFI values in PB CD34+ cells of CALR+ patients expressing the 52-bp deletion (type 1 mutation) or the 5-bp insertion (type 2 mutation). (TIF) [file pone.0220189.s008.tif]
