# Supplementary material for: Constitutive STAT5 phosphorylation in CD34+ cells of patients with primary myelofibrosis: Correlation with driver mutation status and disease severity
Source: PLoS One. 2019 Aug 1;14(8):e0220189. doi: 10.1371/journal.pone.0220189 (PMC6675063; doi:10.1371/journal.pone.0220189)
Supplement: S1 Table — (DOCX) [file pone.0220189.s009.docx]

**S1 Table. Correlations between p-STAT5, TPO induced p-STAT5, and IL6 induced p-STAT3 pathways tested in circulating CD34^+^ cells of patients with PMF and disease parameters.**

|  |  | Constitutive  p-STAT5 | TPO induced  p-STAT5 | IL6 induced  p-STAT3 |
| --- | --- | --- | --- | --- |
|  |  |  |  |  |
| IPSS | R  p | 0.11  0.41 | 0.03  0.8 | 0.17  0.2 |
| DIPSS | R  p | 0.13  0.33 | -0.12  0.4 | 0.21  0.1 |
| WBC count | R  p | -0.04  0.8 | 0.2  0.07 | 0.14  0.3 |
| Monocyte count | R  p | -0.2  0.09 | 0.2  0.17 | -0.03  0.8 |
| Platelet count | R  p | -0.14  0.3 | 0.2  0.13 | -0.02  0.8 |
| Blood blasts | R  p | 0.3  0.14 | -0.26  0.25 | 0.04  0.9 |
| Serum LDH | R  p | 0.16  0.4 | -0.12  0.5 | -0.2  0.3 |
| Spleen size | R  p | 0.23  0.09 | -0.18  0.2 | 0.09  0.5 |
| Degree of BM fibrosis | R  p | 0.17  0.46 | -0.4  0.09 | 0.17  0.4 |
