# Supplementary material for: Constitutive STAT5 phosphorylation in CD34+ cells of patients with primary myelofibrosis: Correlation with driver mutation status and disease severity
Source: PLoS One. 2019 Aug 1;14(8):e0220189. doi: 10.1371/journal.pone.0220189 (PMC6675063; doi:10.1371/journal.pone.0220189)
Supplement: S2 Table — (DOCX) [file pone.0220189.s010.docx]

|  | *JAK2*V617F^+^  Patients |  | Hb | CD34^+^CXCR4^+^  frequency | Chol | CD34^+^ cells  x10^6^L |
| --- | --- | --- | --- | --- | --- | --- |
| TPO induced  p-STAT5 | P-PMF  n=13 | R  p | 0.19  0.52 | -0.15  0.7 | 0.66  0.16 | 0.08  0.81 |
| TPO induced  p-STAT5 | PMF  n=19 | R  p | -0.52  0.022 | -0.3  0.23 | 0.21  0.61 | 0.5  0.033 |
| IL6 induced  p-STAT3 | P-PMF  n=13 | R  p | -0.41  0.16 | -0.28  0.46 | -0.2  0.7 | 0.58  0.06 |
| IL6 induced  p-STAT3 | PMF  n=20 | R  p | -0.31  0.18 | -0.29  0.22 | 0.2  0.61 | 0.49  0.028 |

**S2 Table. Correlations between TPO induced p-STAT5, and IL6 induced p-STAT3 values tested in circulating CD34^+^ cells of *JAK2*V617F^+^ patients with PMF and disease parameters.**
